# MiR‐29b‐3p promotes chondrocyte apoptosis and facilitates the occurrence and development of osteoarthritis by targeting PGRN

**DOI:** 10.1111/jcmm.13237

**Published:** 2017-06-13

**Authors:** Lingqiang Chen, Qin Li, Jing Wang, Song Jin, Hongmei Zheng, Jun Lin, Fang He, Hong Zhang, Sha Ma, Jian Mei, Juan Yu

**Affiliations:** ^1^ Department of Orthopaedics The First Affiliated Hospital of Kunming Medical University Kunming Yunnan Province China; ^2^ Department of Rheumatology The First People's Hospital of Yunnan Province Kunming Yunnan Province China

**Keywords:** MiR‐29b‐3p, Progranulin, Osteoarthritis, Chondrocyte

## Abstract

This study was aimed to explore the role of miR‐29b‐3p and PGRN in chondrocyte apoptosis and the initiation and progress of osteoarthritis (OA). Both miR‐29b‐3p and PGRN were up‐regulated in cartilage tissue from patients with OA. Transfection of miR‐29b‐3p mimic into rat primary chondrocytes and SW1353 chondrosarcoma cells significantly suppressed PGRN expression and release, induced apoptosis, inhibited proliferation and scratch wound closure. By contrast, transfection of miR‐29b‐3p inhibitor exhibited the opposite effects. Moreover, the expression and secretion of cartilaginous degeneration‐related molecules were also altered by miR‐29b‐3p. Luciferase reporter gene assay showed rat GRN mRNA is directly targeted and repressed by miR‐29b‐3p. The fact that recombinant PGRN or shPGRN‐mediated PGRN interference abolished miR‐29b‐3p mimic‐induced cell apoptosis and growth inhibition suggested miR‐29b‐3p affect the cellular functions of chondrocyte through regulating PGRN expression. *In vivo*, joint cavity injection of miR‐29b‐3p antagomir prior to surgical induction of OA significantly suppressed the upregulation of miR‐29b‐3p, whereas further promoted the increased expression of PGRN. Articular chondrocytes apoptosis and cartilage loss in the knee joint of surgically induced OA rats were also ameliorated by the injection of miR‐29b‐3p antagomir, demonstrated by TUNEL and safranin O‐fast green staining. This work showed miR‐29b‐3p facilitates chondrocyte apoptosis and OA by targeting PGRN, and miR‐29b‐3p or PGRN may be the potential target for OA treatments.

## Introduction

OA is a chronic and degenerative disease of the joint (such as knee and hip joint), which is characterized by cartilage loss, subchondral bone changes, synovitis and other joint tissue changes. The clinical features of OA are joint pain, joint swelling, limited motion and joint deformity. The risk factors of OA include ageing, genetics, obesity, abnormal anatomical structure, excessive weight‐bearing and joint injury. Joint replacement is still the main therapy for the treatment of end‐stage OA, and there are still no efficient methods to prevent and combat OA. A substantial body of evidence exists suggesting a role for chondrocyte apoptosis in the occurrence and progression of OA 1. Multiple signalling pathways and cytokines are involved in the apoptosis of chondrocyte in OA, such as nitric oxide 2, Fas 3, p38 MAPK 4, PI3K/Akt 5, IL‐1β 6 and TNF‐α 7.

MicroRNA (miRNA) is a family of 22‐nt non‐coding single‐stranded RNA molecules encoded by endogenous genes involved in the post‐transcriptional regulation of gene expression in various organisms ranging from nematodes to humans. In OA cartilage, mounting evidence suggests that miRNA might play a role in chondrocyte apoptosis. Mohamed M. Abouheif *et al*. demonstrated silencing of miR‐34a could effectively reduce IL‐1β‐induced rat chondrocyte apoptosis *in vitro* 8. Jing Li *et al*. reported miR‐146a contributed to OA pathogenesis by inducing vascular endothelial growth factor levels and chondrocyte apoptosis through targeted inhibition of Smad4 in cartilage 9. In addition, a variety of miRNAs was implicated in the pathogenesis of OA, including miRNA‐142‐3p 10, miRNA‐21 11 and miRNA‐448 12. It was demonstrated that the expression of miR‐29b‐3p was significantly increased at day 1 after ‘destabilisation of the medial meniscus' operation, and gradually increased over time in a certain range 13. Moreover, the level of miR‐29b‐3p in cartilaginous tissue of patients with end‐stage OA was significantly higher than that in the normal cartilage tissue 14, indicating that miR‐29b‐3p may play an important role in the occurrence and development of OA.

Progranulin (PGRN) is a growth factor expressed by a variety of cells types, including epithelial cells, neurons, macrophages and chondrocytes 15 and has been proven to play a role in various physiological processes, such as wound healing, tumourigenesis and neurodegenerative disease. Moreover, PGRN was also implicated in the pathogenesis of OA. It was demonstrated that compared to normal mice, anterior cruciate ligament (ACL) transection‐induced OA was more severe in PGRN deficient mice, and intra‐articular injection of recombinant PGRN significantly prevented the cartilage degeneration 16. Moreover, as one of the downstream molecules of BMP‐2, PGRN can promote the proliferation and differentiation of chondrocyte and cartilage renovation 17. In the process of cartilage formation, PGRN could negatively regulate endoplasmic reticulum stress‐induced chondrocyte apoptosis through TNFR mediated signalling pathway 18.

It was reported that miR‐29b‐3p was a novel posttranscriptional regulator of PGRN expression in frontotemporal dementia (FTD). However, whether miR‐29‐3p could regulate the expression of PGRN in chondrocyte and the effect of miR‐29‐3p on chondrocyte apoptosis and OA process are largely unknown. Therefore, this work was desired to investigate the role of miR‐29‐3p in regulating PGRN expression, chondrocyte apoptosis and pathogenesis of OA.

## Materials and methods

### Patient samples

Cartilages of the femoral head and knee joint were taken from patients with OA (hip OA age 56–79 years, five female and three male; knee OA age 48–74 years, three female and four male). The cartilages from patients (age 45–75 years, four female and five male) with femoral neck fracture and undergoing total joint replacement surgery were used as control. OA was diagnosed by clinical history and detection, and combined with X‐ray findings. Gross pathologic examination was performed at the time of joint replacement. Fracture patients had no joint disease, and the cartilage was free of lesions. This study was allowed by the Ethical Committee of the First Affiliated Hospital of Kunming Medical University, and all patients were provided informed consent.

### Isolation and culture of chondrocytes

The primary chondrocytes were prepared from five newborn SD rats. Briefly, the rats were killed under anaesthesia, and the articular cartilages of the knee joint were removed under sterile condition. After removal of attached muscle, perichondrium and connective tissue, the remaining cartilages were shredded and rinsed for three times with phosphate‐buffered saline (PBS). Then, the cartilages were digested with 2.5 g/l trypsin for 15 min. at 37°C. After removal of the supernatant, the cartilages were washed with PBS and digested with 5–10 times volume of 2 g/l type II collagenase solution at 37°C for 4 hrs. Dissociated cells were passed through a 100 mesh nylon mesh sieve, centrifugalized at 1000 r/min for 8 min. The cells were washed with PBS twice and resuspended in DMEM/F12 supplemented with 10% FBS, 100 U/ml ampicillin and streptomycin. The percentage of dead cells was examined with trypan blue staining. Then, the isolated chondrocytes were plated at 1 × 10^5^ cells/ml in culture flasks after counted and cultured at 37°C, 5% CO_2_ conditions in the incubator. The medium was replaced with fresh medium after 48 hrs. SW1353 chondrosarcoma cells were obtained from the Chinese Academy of Sciences cell bank, and cultured in DMEM supplied with 10% FBS, 2 mM glutamine, 100 U/ml penicillin and streptomycin.

### Transient transfection for functional analysis of miR‐29b‐3p

Primary chondrocytes and SW1353 cells were inoculated into a 6‐well plate at 2.5 × 10^5^ cells per well. After 24 hrs of incubation, cells were transfected for 6 hrs in serum‐ and antibiotic‐free DMEM using Lipofectamine 2000 (Invitrogen, Carlsbad, CA, USA), miR‐29b‐3p mimic at 30 nM (Qiagen, Germantown, MD, USA), miR‐29b‐3p inhibitor at 50 nM (Qiagen) or non‐targeting controls at 30 nM (Qiagen). Medium was replaced with fresh medium after transfection for 6 hrs, and the cells cultured for another 48 hrs before using for further analysis.

### Knock‐down of GRN by shRNA lentivirus interference

Lentiviral vector‐based siRNA plasmids (pILenti‐siRNA‐GFP, Applied Biological Materials, Richmond, BC, Canada) expressing shRNA duplexes that target GRN was transfected into SW1353 chondrosarcoma cells according to the manufacturer's suggested protocol to generate GRN knock‐down chondrosarcoma cells. Cells transfected with the same vector plasmid expressing a scrambled shRNA acted as negative control. The infection efficiency was monitored by the green fluorescent protein (GFP), and puromycin was used for selection of stable cell lines. The two stable chondrosarcoma cell lines generated were identified as: wild‐type and PGRN KD cells, respectively.

### DAPI staining

For cell nucleus morphological information, the cultured cells were washed with PBS for twice, fixed in 4% PFA for 15 min., and stained with 1 g/l DAPI (Beyotime, Nanjing, China) according to the manufacturer's instructions. After removal of DAPI, the cells were washed with PBS for three times and observed under fluorescence microscope. The cells with condensed or fragmented nuclei were supposed to be apoptotic cells.

### Flow cytometry

For cell apoptosis analysis, the cells were digested with EDTA‐free trypsin and washed twice with cold PBS. About 1 × 10^5^ cells were suspended in 500 μl binding buffer containing 2 μl of annexin V‐FITC (20 μg/ml) and 5 ul of PI (50 μg/ml). After incubated for 20 min. in dark at room temperature, the cells were analysed by flow cytometry (BD FACSCalibur, San Jose, CA, USA) at 488 nm. The distribution of cells was analysed using Modfit LT software (Becton‐Dickinson, San Jose, CA, USA) in the flow cytometer within 1 hr of staining. Data from at least 10,000 cells were collected for each data file at a flow rate of 250–300 cells/s. Apoptotic cells were identified as Annexin V‐FITC‐positive and PI‐negative cells. For cell cycle analysis, the cells were digested with EDTA‐free trypsin and washed twice with cold PBS at 48 hrs after transfection. Then the cells were fixed with 70% ethanol and placed overnight at 4°C. After removal of ethanol by centrifugation, the cells were washed with PBS twice and suspended in 1 ml of PBS. After incubation with 10 μg/ml RNase at 37°C for 30 min., and stained with PI (Life Technologies, Carlsbad, CA, USA), the cell cycle was determined using BD FACSCalibur. The data were analysed by the Cell Quest (San Jose, CA, USA) and the FlowJo (FlowJo LLC, Ashland, OR, USA) software to calculate the cell cycle distributions.

### CCK‐8 assay

The effects of miR‐29b‐3p mimic or inhibitor transfection on the proliferation of chondrocytes and SW1353 cells were assessed using a CCK‐8 assay. Following transfection for 12, 24, 36 and 48 hrs, the supernatant was wasted, and 10 μl of CCK‐8 was added to each well. After 2‐hrs incubation at 37°C, the absorbance at 450 nm was recorded.

### Wound‐healing assay

A wound‐healing assay was carried out to investigate the capacity of these cells to repair damage. After the cell fusion into a single layer in six‐well plates, a single scratch wound was generated with a 200‐μl sterile pipette tip. After washed with PBS twice, the cells were cultured with serum free medium for 24 hrs. Then the scratch wounds were observed and photographed under an inverted microscope, and the scratch widths were quantitated with the ImageJ software (V. 1.47; NIH, Bethesda, MD, USA). The data were plotted as the percentage of wound closure, setting the initial scratch width as 100%.

### Luciferase reporter gene assay

The wild‐type and mutant 3′ UTR sequences of Rno GRN were synthesized by GenePharma (Shanghai, China) and subcloned into the pGL3 promoter vector containing the luciferase reporter (Promega, Madison, WI, USA). The recombinant plasmids were respectively named as, pGL3‐GRN 3′ UTR‐WT, pGL3‐GRN 3′ UTR‐MU1 and pGL3‐GRN 3′ UTR‐MU2. SW1353 or HEK293T cells were seeded in 24‐well plates. When the cells reached about 70% confluences, each recombinant plasmid (200 ng) and miR‐29b‐3p mimic (200 ng) were cotransfected into the cells with Lipofectamine 2000 (Invitrogen) according to the manufacturer's direction. The pGL3 promoter vector (200 ng) was used as the control. To normalize the activity of fly luciferase, we cotransfected the pRL‐SV40 Renilla luciferase control reporter vector (Promega) into SW1353 or HEK293T cells. Experiments were performed in quadruplicate and repeated at least three times.

### Surgical induction of OA

All the animal procedures were approved by the by the Animal Care and Use Committee of the First Affiliated Hospital of Kunming Medical University. Thirty 10‐week‐old SD male rats (Shanghai SLAC laboratory animal Co., Ltd., Shanghai, China) were randomly divided into three groups: Sham operation group (Sham), OA negative control group (OA/NC) and OA miR‐29b‐3p antagomir treatment group (OA/Antagomir). The rats in OA/NC and OA/Antagomir were respectively received a one‐time injection of 800 pmol of miR‐29b‐3p negative control or miR‐29b‐3p antagomir into the joint 6 hrs before the operation and were executed at 2 days, 4 days, 3 weeks, 4 weeks and 6 weeks post‐operatively, in which, nine rats were randomly chosen and killed in each group per time. The OA model was established by surgery following a previous report 19. Briefly, the anterior/posterior cruciate ligament, medial collateral ligament and meniscus medialis of the right hindlimb of the rat were removed to cause the joint instability, which resulted in the increase of the wear of the articular surface and the degeneration of the articular cartilage. Rats in sham group were only exposed the surface of the articular cartilage and were killed at 6 weeks post‐operatively. The whole knee joints were collected for follow‐up analysis.

### TUNEL and safranin O‐fast green staining

The whole knee joints were placed in 4% poly formaldehyde and fixed for 24 hrs. After conventional dehydrated, transparent and paraffin embedded, the joints were sliced into 5 μm continuous sections. TUNEL and safranin O‐fast green staining were performed using the *In Situ* Cell Death Detection kit, POD (Roche Diagnostics GmbH, Mannheim, Germany) and Safranin O/Fast Green staining kit (ICH World, Woodstock, MD, USA) in accordance with the manufacturer's introduction. For histomorphometric analysis, the Safranin O‐positive cartilage area and thickness were quantified by Image‐Pro Plus 6.0 (Media Cybernetics, Bethesda, MD, USA). The Safranin O/Fast Green stained sections were used to examine the OA scoring using a modified Mankin's Score (mRS) system 20.

### RNA isolation and qRT–PCR

Total RNAs were prepared from flash frozen tissue and cells using TRIZOL^©^ reagent (Invitrogen) in accordance with manufacturer's instructions. Equal amount of RNA was then reverse‐transcribed into cDNA using random hexamer primers with the TaqMan miRNA reverse transcription kit (Applied Biosystems, Foster City, CA, USA). The qRT–PCR was carried out using SYBR^®^ Green Real‐time PCR Master Mix (Toyobo Co. Ltd., Osaka, Japan) using the following conditions: 94°C for 5 min., and followed by 30 cycles of 94°C for 30 sec., 58–61°C for 30 sec. depending on the primers, and 72°C for 2 min. The mRNA level of β‐actin (for mRNAs) or U6 (for miRNAs) was used as an internal control, and the relative gene expression levels were calculated using the 2^−ΔΔCt^ method. Each gene was analysed in triplicate. The primer sequences were listed in Table S1.

### Western blotting analysis

The cartilage tissues were ground under liquid nitrogen, and the cell samples were washed with pre‐cooling PBS. Then, the samples were lysed in ice‐cold RIPA lysis buffer (Beyotime, Jiangsu, China), and the total protein content was measured using a BCA protein assay kit (Applygen, Beijing, China). All samples were treated with a mixture of proteinase‐free chondroitinase ABC, keratanase and keratanase II (Sigma‐Aldrich, St. Louis, MO, USA) to remove GAG before electrophoretic separation. Equal amounts of total proteins were separated by SDS‐PAGE using 10% gels and transferred onto PVDF membranes (Thermo Fisher Scientific, Waltham, MA, USA). After blocking for 30 min. at room temp in blocking solution containing 5% non‐fat milk, the membranes were incubated overnight at 4°C with primary antibody. After several washes, the membranes were incubated with an appropriate HRP‐conjugated secondary antibody for 1 hr at room temperature. The proteins bands were visualized using ECL kits (Amersham), and the optical density of the protein bands was quantified using the ImageJ software, using GAPDH as an internal control. The primary antibodies were as follows: Rabbit anti‐human PGRN antibody (1:1000, ab108608; Abcam, Cambridge, UK), Rabbit anti‐rat PGRN antibody (1:1000, ab191211; Abcam), Rabbit anti‐human Cleaved caspase‐3 antibody (1:500, ab 32042; Abcam), Rabbit anti‐rat caspase‐3 antibody (1:500, ab13847; Abcam), Anti‐Bax antibody (1:1000, ab32503; Abcam), Anti‐Bcl‐2 antibody (1:1000, ab201566; Abcam), Anti‐ADAMTS‐5 antibody (1:250, ab41037; Abcam), Anti‐ADAMTS‐7 antibody (1:1000, ab203027; Abcam), Anti‐MMP‐1 antibody (1:1000, ab201566; Proteintech, Chicago, IL, USA), Anti‐MMP‐13 antibody (1:1000, ab80734; Abcam), Anti‐COMP antibody (1:100, DMABT‐H12224; Creative Diagnostics, Shirley, NY, USA), Anti‐COL II antibody (1:1000, ab188570; Abcam), Anti‐Aggrecan antibody (1:5000, ab78292; Abcam), Anti‐COL X antibody (1:1000, ab182563; Abcam), Anti‐IL‐1β antibody (1:1000, ab9722; Abcam), Anti‐TNF‐α antibody (1:100, ab199013; Abcam), Anti‐GAPDH antibody (1:1000, ab8245; Abcam).

### ELISA assays

The PGRN, MMP‐1, MMP‐13, COL II and COL X in the culture medium of the rat primary chondrocytes and SW‐1353 cells were measured using ELISA kits according to the manufacturer's instructions. The ELISA kits used were listed as follows: Rat PGRN kit (YS01226B; Y‐J Biological, Shanghai, China), Rat MMP‐13 kit (CSB‐E07412r‐CSB, Cusabio Biotech, Wuhan, China), Rat Collagen II kit (LS‐F11156, LifeSpan BioSciences, Seattle, WA, USA), Rat COL X kit (abx155379, Abbexa Ltd, Cambridge, UK), Human PGRN kit (R&D Systems, Minneapolis, MN, USA), Human MMP‐1 kit (ab100603; Abcam), Human MMP‐13 kit (ab100605; Abcam), Human COL II kit (LS‐F6389; LifeSpan BioSciences), Human COL X kit (LS‐F13131, LifeSpan BioSciences). For the analysis of COL II (COL2A1) and COL X (COL10A1) protein levels, pepsin was used to prevent the collagen deposition in the media.

### Statistical analysis

All data were analysed using SPSS13.0 software (SPSS, Inc., Chicago, IL, USA) and presented as means ± S.E.M.. Statistical significance between different groups was compared with student's *t*‐test, and *P* < 0.05 or *P* < 0.01 was considered as statistically significant. All assays were performed in triplicate.

## Results

### MiR‐29b‐3p and PGRN were both overexpressed in OA

**Figure 1:**
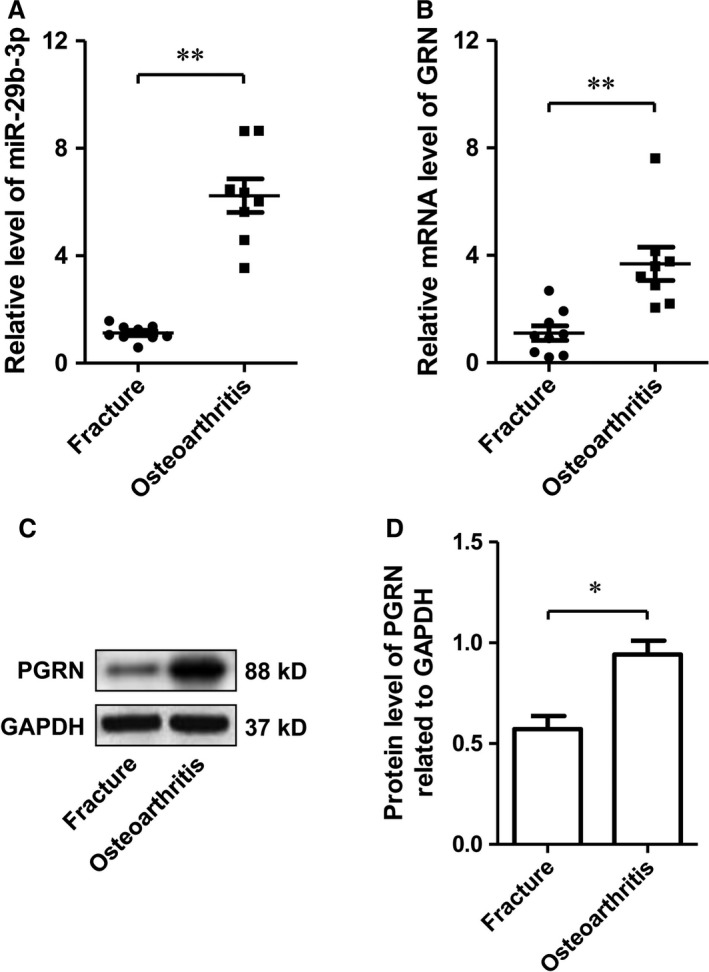
Expression levels of miR‐29b‐3p and PGRN in cartilage tissues from osteoarthritis and fracture patients. **A**. Relative miR‐29b‐3p levels in clinical specimens detected by qRT–PCR. **B**. Relative expression of GRN at transcriptional level detected by qRT–PCR. **C‐D**. Protein level of PGRN in clinical specimens measured by Western blotting and the semi‐quantitative densitometry results. *P < 0.05, **P < 0.01, Fracture *versus* Osteoarthritis.

To verify that MiR‐29b‐3p and PGRN were involved in the pathological process of OA, we explored the expression of MiR‐29b‐3p and PGRN in the cartilage of patients with OA. The results showed that the expression of miR‐29b‐3p and PGRN in patients with OA was significantly higher than that in patients without OA (Fig. 1), suggesting that miR‐29b‐3p and PGRN may be involved in the pathogenesis of OA.

### PGRN was negatively regulated by miR‐29b‐3p in rat primary chondrocytes and SW‐1353 chondrosarcoma cells

**Figure 2:**
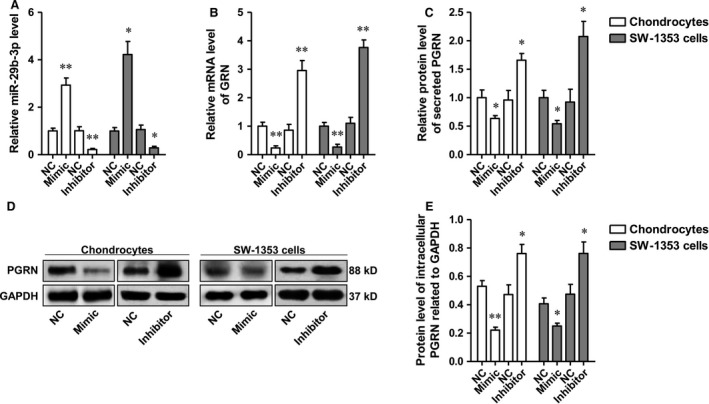
miR‐29b‐3p negatively regulates PGRN in primary cultured rat chondrocytes and SW‐1353 human chondrosarcoma cells. **A‐B**. miR‐29b‐3p and GRN mRNA levels in the two types of cells after transfected with miR‐29b‐3p mimic or inhibitor were determined by qRT–PCR. **C**. After transfection with the mimic or the inhibitor, ELISA was performed to detect the secretion of PGRN. **D‐E**. Representative Western blots of intracellular PGRN of the primary chondrocytes and SW‐1353 cells and the corresponding semi‐quantitative densitometry results shown in histogram. *P < 0.05, **P < 0.01, compared with NC (negative control).

The rat primary chondrocytes and SW‐1353 chondrosarcoma cells were transfected with miR‐29b‐3p mimic or inhibitor, respectively, and the levels of miR‐29 family were determined using qRT–PCR. The data showed that miR‐29b‐3p level was significantly altered by miR‐29b‐3p mimic or the inhibitor, which showed no significant influence on the levels of miR‐29a‐3p and miR‐29c‐3p (Fig. 2A and Fig. S1). After confirmation of the specific regulation effect, the influence of miR‐29b‐3p on PGRN expression and release was determined. The results suggested the transcription, translation and secretion of PGRN by primary chondrocytes, and chondrosarcoma cells were negatively regulated by miR‐29b‐3p (Fig. 2B–E).

### MiR‐29b‐3p accelerated apoptosis of rat primary chondrocytes and SW‐1353 chondrosarcoma cells

**Figure 3:**
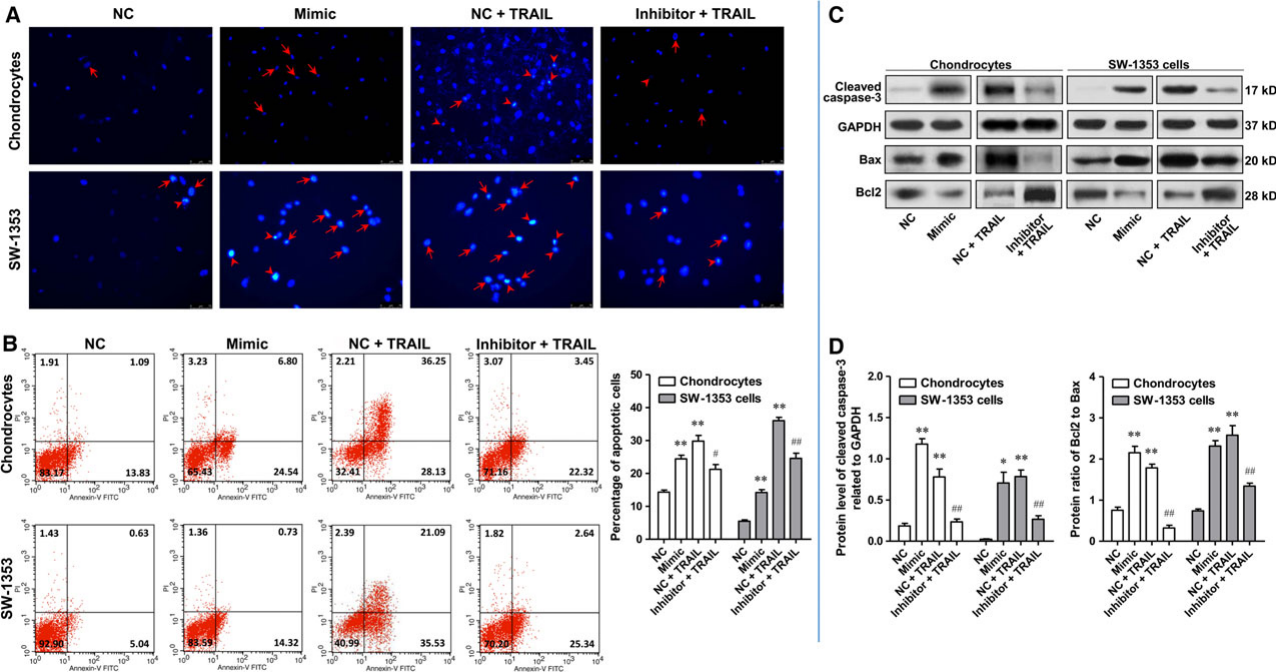
miR‐29b‐3p accelerated apoptosis of primary cultured rat chondrocytes and SW‐1353 cells. **A**. Photomicrographs (400 × ) of the nucleus the cells stained with DAPI. **B**. Cell apoptosis of the chondrocytes and chondrosarcoma cell line analysed using flow cytometry (FCM). The histogram showed the results based on three paralleled experiments. **C‐D**. The protein level of cleaved caspase‐3, Bax and Bcl2 measured with Western blotting. The histogram showed the relative level of cleaved caspase‐3 and the ratio of Bax to Bcl2. *P < 0.05, **P < 0.01, compared with NC; ^#^P < 0.05, ^##^P < 0.01, compared with TRAIL treatment group.

A substantial body of evidence exists suggesting OA significantly correlates with chondrocyte apoptosis. Therefore, the effect of MiR‐29b‐3p on cell apoptosis was investigated in rat primary chondrocytes and SW‐1353 cells. After transfection with miR‐29b‐3p mimic or miR‐29b‐3p inhibitor for 48 hrs, the apoptosis of chondrocytes and SW‐1353 cells was determined using PADI staining, flow cytometry and the Western blotting analysis of cleaved caspase‐3, Bax and Bcl2. Please note that due to the low level of apoptosis in natural state, it is difficult to observe the anti‐apoptotic effect of miR‐29b‐3p inhibitor. Therefore, in the verification of the anti‐apoptotic effect of miR‐29b‐3p inhibitor, the apoptosis was induced by TRAIL after transfection with miR‐29b‐3p inhibitor. DAPI staining is a commonly used method for detection of apoptosis. Under the fluorescence microscope, early apoptosis cells showed nuclear enrichment and stain deepen, or the nuclear chromatin clustered on the inner border of karyotheca. In the late apoptotic cell, the cell nucleus is broken into fragments of different sizes and is surrounded by the cell membrane. In Figure 3A, it appeared that the number of apoptotic cells in mimic treatment group was notably more than that in negative control (NC) group, and transfection with miR‐29b‐3p inhibitor notably suppressed the apoptosis induced by TRAIL in rat primary chondrocytes and SW‐1353 chondrosarcoma cells. This was reinforced by the experimental results of flow cytometry. The percentages of early and late apoptotic cells in the mimic treatment group were significantly higher than those in the NC group. Compared with the TRAIL group, miR‐29b‐3p inhibitor could reduce the proportion of early and late apoptotic cells (Fig. 3B). Cleaved caspase‐3 can lead to the loss of function of poly ADP‐ribose polymerase (PARP), which is closely related to DNA repair and gene integrity monitoring, and plays an important role in cell apoptosis. Bax is found to interact with Bcl2 to form heterodimer or with itself to form homodimer, and increased Bax homodimer often lead to apoptosis. Transfection with miR‐29b‐3p mimic dramatically increased the protein level of cleaved caspase‐3 and the protein ratio of Bcl2 to Bax in both primary chondrocytes and SW‐1353 cells, and transfection with miR‐29b‐3p inhibitor effectively suppressed the increase of these two indexes induced by TRAIL (Fig. 3C and D).

### MiR‐29b‐3p suppressed proliferation of rat primary chondrocytes and SW‐1353 chondrosarcoma cells, induced their cell cycle arrest and inhibited the scratch wound closure

**Figure 4:**
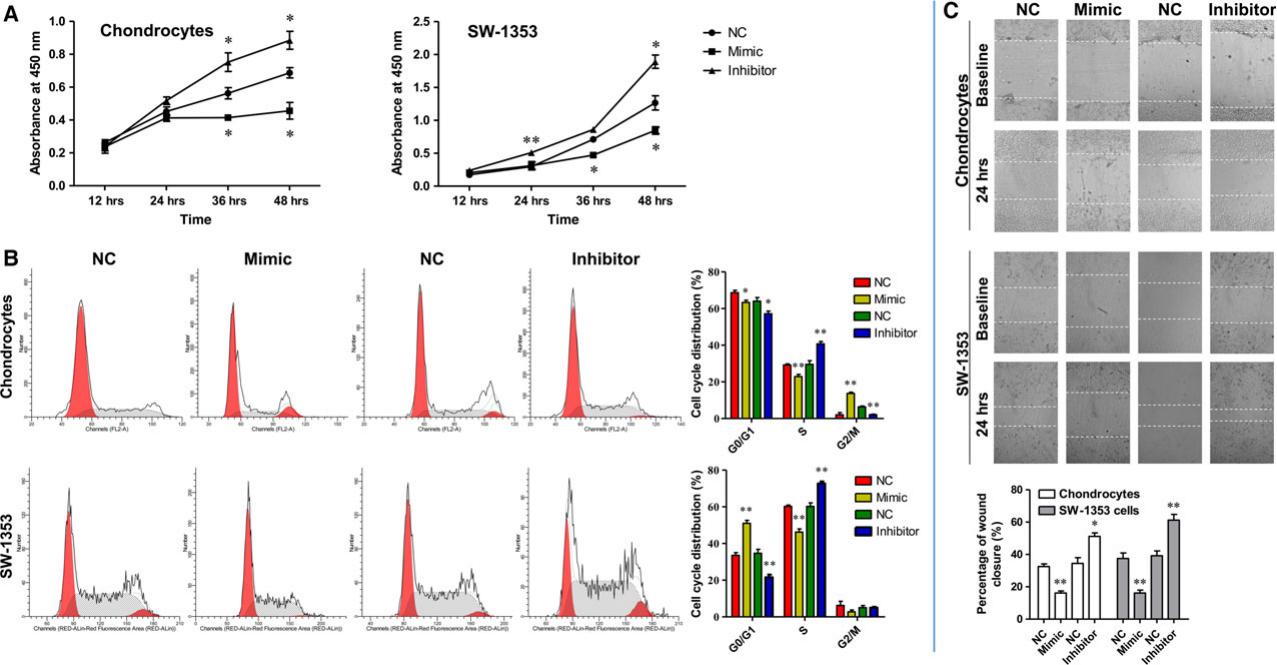
miR‐29b‐3p inhibited the progressions of primary rat chondrocytes and SW‐1353 human chondrosarcoma cells. **A**. miR‐29b‐3p mimic and inhibitor significantly altered the proliferation of the chondrocytes and SW‐1353 cells. **B**. miR‐29b‐3p mimic‐induced cell cycle arrest and miR‐29b‐3p inhibitor caused accelerated cell division. **C**. miR‐29b‐3p mimic hindered the closure of scratch wound, while the inhibitor promoted the healing of scratch wound. *P < 0.05, **P < 0.01, compared with NC.

Promoting chondrocytes differentiation and proliferation by gene regulation may help to achieve the cure of OA. Thus, the effects of MiR‐29b‐3p on cell proliferation and cycle were also evaluated in rat primary chondrocytes and SW‐1353 chondrosarcoma cells using CCK‐8 assay and flow cytometry. Transfection with miR‐29b‐3p mimic significantly inhibited the cell proliferation, and induced cell cycle arrest in G2/M phase in primary rat chondrocytes and in G0/G1 phase in SW‐1353 chondrosarcoma cells (Fig. 4A and B). In contrast, transfection with miR‐29b‐3p inhibitor effectively promoted the proliferation of these two kinds of cells and the entrance of cells from G0/G1 to S phase. In addition, a wound‐healing assay was carried out to investigate the effect of miR‐29b‐3p on the capacity of these cells to repair damage. The results showed that miR‐29b‐3p mimic prevented the repair of cell scratch injury, while miR‐29b‐3p inhibitor promoted the repair of cells after scratch injury (Fig. 4C and D). Taken together, MiR‐29b‐3p suppressed proliferation of rat primary chondrocytes and SW‐1353 chondrosarcoma cells induced their cell cycle arrest and inhibited the scratch wound closure, which indicated that blocking of miR‐29b‐3p might contribute to cartilage repair in OA.

### MiR‐29b‐3p altered the secretion of cartilaginous degeneration‐related molecules and had a direct binding site at 3′ UTR of rat GRN mRNA

**Figure 5:**
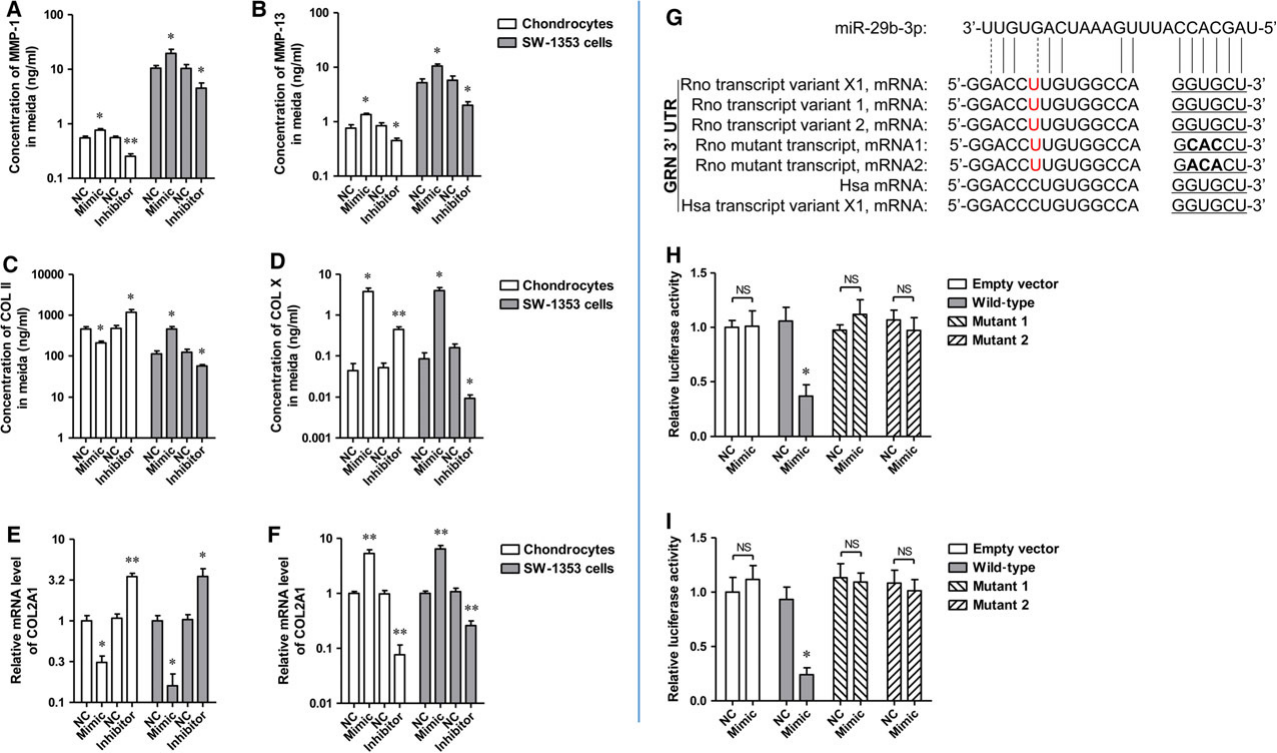
miR‐29b‐3p regulated cartilaginous degeneration‐related molecules, including MMP‐1, MMP‐13, COL II and COL X, and direct targeted at 3′ UTR of rat GRN (rGRN) mRNA. **A‐D**. Concentrations of secreted MMP‐1, MMP‐13, COL II and COL X in the media of rat primary chondrocytes or SW‐1353 human chondrosarcoma cells after transfection with the miR‐29b‐3p mimic or the inhibitor measured by ELISA assay. **E, F**. mRNA expression of COL2A1 and COL10A1 of rat primary chondrocytes or SW‐1353 human chondrosarcoma cells after transfection with the miR‐29b‐3p mimic or the inhibitor measured by qRT–PCR assay. **G**. Schematic representation of miR‐29b‐3p's predicted binding site in the 3′UTR of rGRN mRNAs. **H, I**. The relative luciferase activity after the cotransfection of miR‐29b‐3p mimic and 3′ UTR of GRN mRNA into HEK293 and SW1353 cells respectively. *P < 0.05, **P < 0.01, compared with NC (negative control). NS: not significant.

The imbalance of extracellular matrix (ECM) synthesis and degradation of articular chondrocytes is one of the important reasons for the degeneration of cartilage. MMPs are the most important protein hydrolysis system of ECM degradation. MMP‐1 and MMP‐13 can directly degrade Collagen Type II (COL II), which is the most abundant collagen in ECM. Collagen Type X (COL X) is synthesized by hypertrophic chondrocytes, which can be used as a specific marker for severe OA. Transfection with miR‐29b‐3p mimic significantly increased the production of MMP‐1, ‐13 and COL X by rat primary chondrocytes and SW‐1353 cells, whereas reduced COL II transcription and secretion, and transfection with miR‐29b‐3p inhibitor has the opposite influences on the synthesis and release of MMP‐1, ‐13, COL II and COL X (Fig. 5A‐F).

It has been demonstrated that the 3′ UTR of hPGRN mRNA contains a miR‐29b‐3p binding site, and that miR‐29b‐3p and its binding sites in PGRN 3′ UTRs are highly conserved 21. Therefore, it is completely possible that the 3′ UTR of Rno PGRN mRNA also contains a miR‐29b‐3p binding site. As predicted by a miRNA target prediction software, there was a putative binding site for miR‐29b‐3p in both rno GRN 3′UTR and hsa GRN 3′ UTR (Fig. 5G). To validate the interaction between miR‐29b‐3p and rno GRN 3′ UTR, luciferase reporter assay was performed in HEK293T and SW1353 cells. Cotransfection of the recombinant luciferase vector and miR‐29b‐3p mimic remarkably decreased luciferase expression than an empty vector that lacked Rno GRN3′ UTR sequence (Fig. 5H and I). To investigate whether the interaction between miR‐29b‐3p and Rno GRN 3′ UTR is direct or indirect, we mutated the miR‐29b‐3p binding site in Rno GRN 3′ UTR in which GTG was changed to CAC or ACA (Fig. 5G). After cotransfection of the luciferase vector with the mutant rno GRN 3′ UTR and miR‐29b‐3p mimics, it was found that miR‐29b‐3p mimics failed to suppress luciferase expression (Fig. 5H and I), indicating that miR‐29b‐3p interacts directly with the binding site in rno GRN 3′ UTR to regulate luciferase reporter expression.

### PGRN participated in the miR‐29b‐3p mimic‐induced apoptosis in chondrocytes

**Figure 6:**
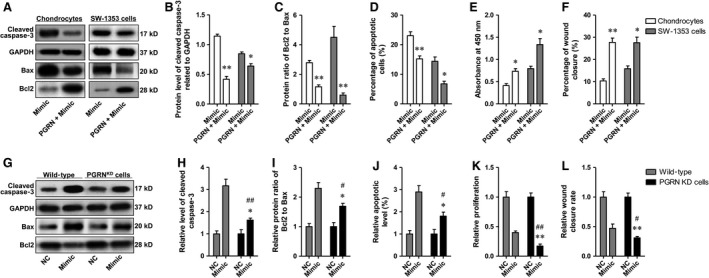
Recombinant PGRN or shPGRN‐mediated PGRN interference antagonized miR‐29b‐3p mimic‐induced cell apoptosis and growth inhibition. **A‐C/G‐I**. The protein level of cleaved caspase‐3, Bax and Bcl2 measured with Western blotting. **D/J**. Percentage of apoptotic cells determined by FCM after treatment with miR‐29b‐3p mimic or combination of PGRN and the mimic. **E/K**. Cell proliferation measured with CCK‐8 kit. **F/L**. Percentage of wound closure measured on the basis of scratch wound assay. *P < 0.05, **P < 0.01, compared with NC (negative control); ^#^P < 0.05, ^##^P < 0.01, compared with Wild‐type mimic group.

As miR‐29b‐3p could suppress the production and secretion of PGRN, we speculated that the effect of miR‐29b‐3p mimic on the apoptosis, proliferation and migration of primary rat chondrocytes and SW‐1353 chondrosarcoma cells was due to PGRN deficient. To test this, we transfected the primary chondrocytes and SW‐1353 cells with miR‐29b‐3p mimics in the presence of recombinant PGRN (100 ng/ml). After cultured for 48 hrs, apoptosis and proliferation were detected using Western blotting analysis, flow cytometry and CCK‐8 assay. Wound‐healing assays were also performed to detect the capacity of these cells to repair damage. Both recombinant rat and human PGRN reduced the up‐regulated cleaved caspase‐3 and Bax/Bcl2 ratio induced by miR‐29b‐3p mimic in the corresponding cells, and may therefore cause the decrease of apoptotic cells (Fig. 6A‐D). The recombinant rat and human PGRN eliminated the restrain of cell proliferation by the mimic (Fig. 6E), and they also accelerated the wound closure, compared with the cells only treated with miR‐29b‐3p mimic (Fig. 6F).

To exclude the inhibitive effect of PGRN itself on apoptosis, the basal expression of PGRN in SW‐1353 chondrosarcoma cells was silenced using shPGRN prior to transfection of miR‐29b‐3p mimic, and the efficiency was determined using qRT–PCR and Western blotting. The results showed that lenti‐shRNA‐mediated PGRN knock‐down was sufficient to cause a 90.5% loss of mRNA expression and a 93.0% loss of protein expression (Fig. S2 A‐C). In addition, transfection with miR‐29b‐3p mimic could not effectively reduce the mRNA and protein expressions of GRN in GRN knock‐down cells as it did in wild‐type cells (Fig. S2 D‐F), and the secreted PGRN in the media was not detectable (by ELISA, data not shown). The data indicated that in PGRN knock‐down cells miR‐29b‐3p mimic could not affect cellular functions through regulating PGRN expression. Afterwards, apoptosis, proliferation and wound closure were determined again in SW‐1353 wild‐type cells and PGRN KD SW‐1353 cells, and the results showed that shPGRN‐mediated PGRN interference partly abolished the effect of miR‐29b‐3p mimic on apoptosis, proliferation and wound closure (Fig. 6G–L), suggesting that miR‐29b‐3p affect the cellular functions of chondrosarcoma cells through regulating PGRN expression.

### MiR‐29b‐3p antagomir reduced the apoptosis of articular chondrocytes in the knee joint of surgically induced rat OA model

**Figure 7:**
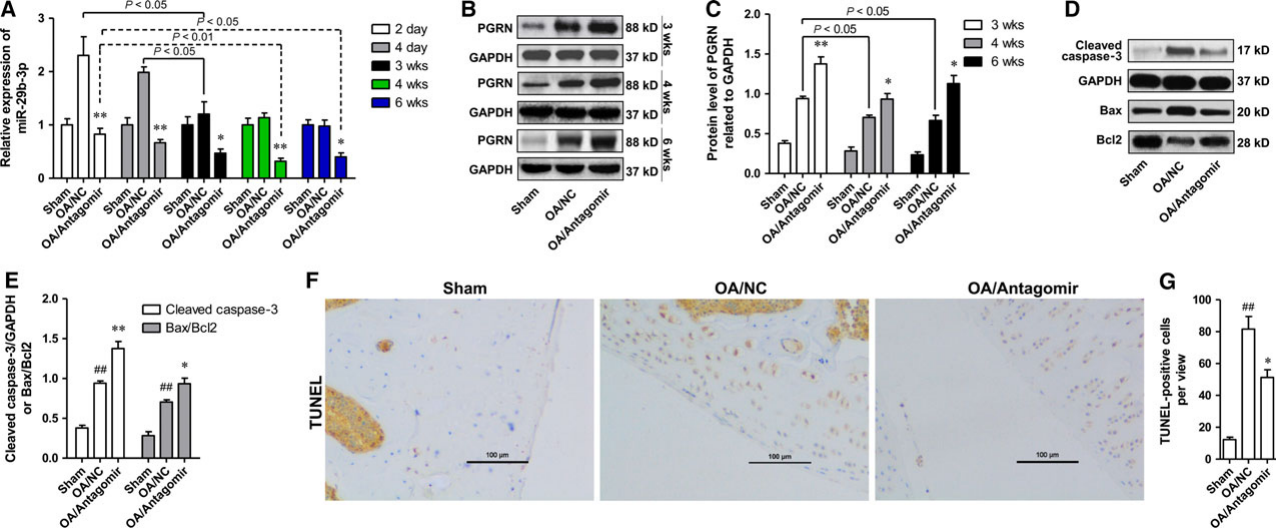
Knock‐down of miR‐29b‐3p resulted in decreased cell apoptosis of articular chondrocytes of osteoarthritic rats. **A**. 2, 4 days, 3, 4 or 6 weeks after surgery, miR‐29b‐3p level in the tibial cartilage was detected by qRT–PCR. **B‐C**. Three, four or six weeks after surgery, PGRN in the tibial cartilage was measured with Western blotting, and the semi‐quantitative results were shown in the histogram. **D‐E**. Cleavage of caspase‐3 and the Bax/Bcl2 ratio in the cartilage were measured with Western blotting, and the histogram presented the semi‐quantitative results. **F‐G**. Apoptotic chondrocytes in the cartilage were identified by TUNEL analysis, and cell counting results of the positive stained were presented in the histogram. **P < 0.01, *P < 0.05, compared with OA/NC group; ^##^P < 0.01, ^#^P < 0.05, compared with Sham group.

Based on the results of *in vitro* investigations, we speculated that knock‐down of miR‐29b‐3p may help reduce the apoptosis of chondrocytes in OA *in vivo*. To test this hypothesis, the rats were treated with miR‐29b‐3p antagomir prior to induction of OA model by surgery, and the apoptosis of chondrocytes was detected at 6 weeks after surgery. The specificity of the antagomir to miR‐29b‐3p was determined *in vitro*, and miR‐29b‐3p antagomir showed no significantly influence on the levels of miR‐29a‐3p and miR‐29c‐3p (Fig. S1). The miR‐29b‐3p level in OA/NC group showed no significant changes at 24 hrs after the surgery (data not shown), whereas peaked rapidly after 2 days and gradually decreased with time (Fig. 7A). Intra‐articular injection of miR‐29b‐3p antagomir significantly suppressed OA‐induced up‐regulation of miR‐29b‐3p throughout the experiment period (Fig. 7A). The PGRN level in cartilage tissue of the OA/NC group was higher than that of the sham operation group (Sham) at 3, 4 and 6 weeks post‐operation, which was significantly up‐regulated by miR‐29b‐3p antagomir pretreatment (Fig. 7B and C). The level of apoptosis in tibial cartilage was measured by determining the level of Cleaved caspase‐3, the ratio protein of Bax/Bcl2 using Western blotting and TUNEL staining. The results showed that the amount of cleaved caspase‐3, the Bax/Bcl2 ratio and the number of TUNEL‐positive cells were significantly increased after the induction of OA, and pretreatment with miR‐29b‐3p antagomir could significantly inhibit the increase of cell apoptosis (Fig. 7D‐G).

### MiR‐29b‐3p antagomir impeded the loss of cartilage in the knee joint of osteoarthritic rats

**Figure 8:**
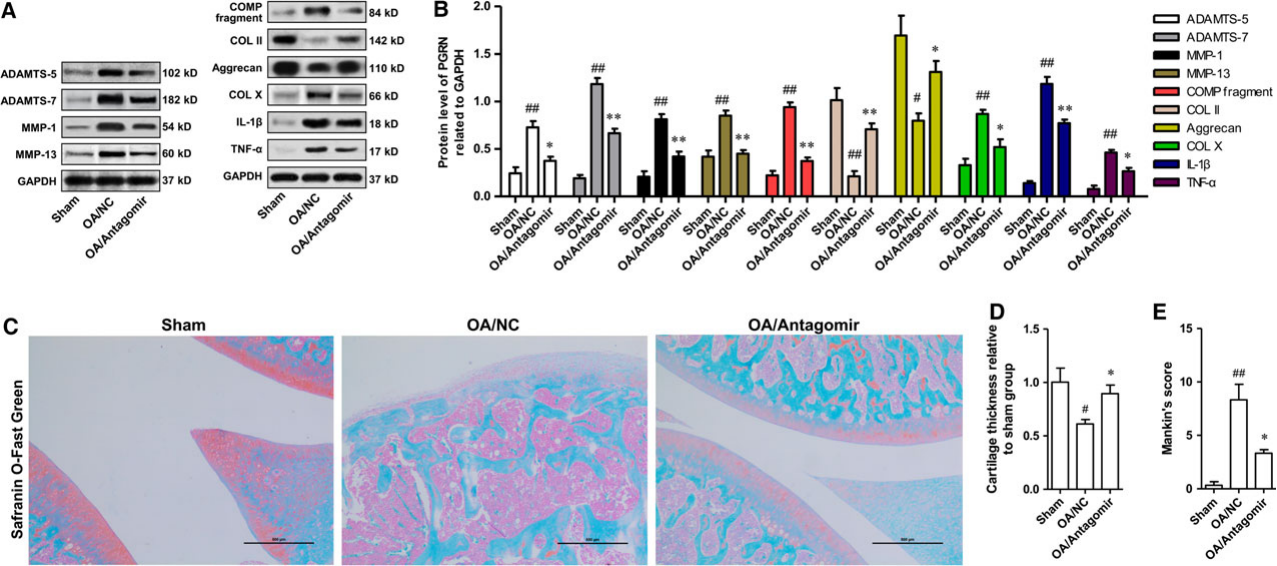
Knock‐down of miR‐29b‐3p attenuated cartilage loss in knee joint of osteoarthritic rats. **A‐B**. Protein levels of catabolic biomarkers (ADAMTS‐5, ‐7, MMP‐1, ‐13 and COMP fragment), anabolic biomarkers (COL II and Aggrecan), the hypertrophic chondrocyte marker (COL X) and inflammatory cytokines (IL‐1β and TNF‐α). **C**. Representative safranin O‐fast green staining of the knee joint from each group. **D**. Cartilage thickness of tibia measured by histomorphometry. **E**. Analysis of degenerative changes by Mankin's scoring. **F**. **P < 0.01, *P < 0.05, compared with OA/NC group; ^##^P < 0.01, ^#^P < 0.05, compared with Sham group.

The main pathological feature of OA is the imbalance between synthesis and degradation of ECM, which leads to the reduction of cartilage matrix. Cartilage that covers the surface of the joint can even be completely consumed. After confirmation of the anti‐chondrocyte apoptosis function of MiR‐29b‐3p antagomir in OA, we determined the effect of miR‐29b‐3p antagomir on cartilage loss. After induction of OA, the protein expression levels of ADAMTS‐5, ADAMTS‐7, MMP‐1 and MMP‐13 were up‐regulated, causing the loss of articular cartilage, which was characterized by increased degeneration of COMP, COL II and Aggrecan. MiR‐29b‐3p Antagomir could inhibit the production of matrix‐degrading enzymes, and reduce the degradation of COMP, COL II and Aggrecan (Fig. 8A and B). Moreover, miR‐29b‐3p antagomir could also suppress the production of inflammatory factors (TNF‐α and IL‐1β) in OA. The influence of miR‐29b‐3p antagomir on cartilage loss in OA was further validated by safranin O‐fast green staining. The mice from the sham operation group exhibited complete structure of knee‐joint cartilage with uniformly stained Safranin O (Fig. 8C). However, mice in the OA/NC group presented symptoms of knee‐joint OA, including the formation of surface fissures, drastically reduced Safranin O staining at the superficial layer and polymorphic layer and the appearance of hypertrophic chondrocytes at the deeper layers (Fig. 7C). Histomorphometric analysis showed that the thickness of knee‐joint cartilage was extensively decreased in mice from OA/NC group (Fig. 8D). In addition, the Mankin's score of mice in OA/NC group was notably higher than that in sham group (Fig. 8E). In contrast, pretreatment with miR‐29b‐3p antagomir could considerably inhibit the loss of articular cartilage and reduce the Mankin's score, demonstrating that the knee‐joint cartilage degeneration was notably restrained in OA mice by miR‐29b‐3p antagomir.

## Discussion

OA is a chronic joint disease that is characterized by degeneration of articular cartilage and bone regeneration of subchondral bone and joint margins. OA often occurs in the facet joints of knee, hip, elbow and hand and predominantly affects articular cartilage, subchondral bone, ligaments and capsule, synovial membrane and surrounding muscles. At present, it is generally considered that OA is caused by a variety of factors, including genetic heritage, biomechanical factors, cartilage nutritional, metabolic abnormalities, chondrocyte apoptosis, cytokines and free radicals.

MiRNAs are a family of endogenous, non‐coding single chain small RNA, with about 22–23 nucleotides length, and widely exists in plants, animals and microbes. Over the past decade, mounting evidence shows that a variety of miRNAs was involved in the occurrence and development of OA. It was identified that the expression of miR‐483, miR‐22, miR‐377, miR‐103, miR‐16, miR‐223, miR‐30b, miR‐23b and miR‐509 was up‐regulated in osteoarthritic cartilage of human knee join compared to normal cartilage, but the expression of miR‐29a, miR‐140, miR‐25, miR‐337, miR‐210, miR‐26a and miR‐373 was down‐regulated 22. Dimitrios Iliopoulos *et al*. demonstrated in OA miR‐22 directly inhibited the expression of BMP‐7 and PPARA, which led to the increased expression of IL‐1 and MMP‐13 and subsequent chondrocytes degeneration and matrix degradation 23. Yamasaki *et al*. reported that inflammatory cytokines induced miR‐146a expression might negatively regulate catabolic factors such as MMP13 expression through miR‐146‐negative feedback including down‐regulation of IRAK1 and TRAF6 in early OA cartilage 24. A study by Bo Yang *et al*. showed that miR‐145 directly regulated mothers against decapentaplegic homologue 3 (Smad3), a key factor in maintaining chondrocyte homeostasis, which resulted in a change of its downstream target gene expression as well as IL‐1β‐induced ECM degradation in OA chondrocytes 25. In addition, some other miRNAs were also reported to be involved in the progression of OA, such as miR‐140 26, miR‐483 22.

The miR‐29 family contains miR‐29a, miR‐29b, and miR‐29c, differing only in two or three bases, miR‐29a and miR‐29b1 as well as miR‐29c and miR‐29b2. It was demonstrated that the expression of the miR‐29 family is up‐regulated in cartilage during OA, which was repressed by SOX9 and regulated by TGF‐β1 and IL‐1 in chondrocytes. The miR‐29 family negatively regulates Smad, NFκB and canonical Wnt signalling and direct targets several Wnt‐related genes 13. In addition, decreased expression of miRNA‐29 family has been demonstrated to be necessary for chondrogenic differentiation of mesenchymal stem cells and cartilage and *in vivo* cartilage/bone formation by targeting FOXO3A 27, 28. In the present study, we found the expression of miR‐29b‐3p was dramatically up‐regulated in cartilage tissue from OA patients compared with that from fracture patients, which raise the possibility that miR‐29b‐3p was involved in the pathogenesis of OA.

To identify the function of miR‐29b‐3p in OA, we first determined the effect of overexpression or knock‐down of miR‐29b‐3p on the cell apoptosis, proliferation, cycle and scratch wound closure in rat primary chondrocytes and SW‐1353 human chondrosarcoma cells *in vitro* by transfection with miR‐29b‐3p mimic or inhibitor. After confirmation of successful transfection by qRT–PCR, it was found that increased expression of miR‐29b‐3p significantly promoted apoptosis, inhibited the proliferation and induced cell cycle arrest in both cell types, whereas the down‐regulation of miR‐29b‐3p resulted in significant repression of apoptosis and promoted the proliferation of these cells and the entrance from G0/G1 to S phase. In addition, results from the wound‐healing assay revealed that transfection of miR‐29b‐3p mimic significantly prevented the repair of cell scratch injury, suggesting the reduction of damage repair capacity of these cell types. Although no direct evidence shows miR‐29b‐3p could accelerate chondrocyte apoptosis at present, the inducing effect of miR‐29 family on apoptosis and cell cycle arrest has been described in a variety of tumour cell types and some normal cell types, including chronic myelogenous leukaemia cells 29, glioblastoma cells 30, neuronal cell 31 and multiple myeloma cells 32, and the mechanisms were usually associated with the regulation of BCR/ABL1, Mcl‐1, Bcl2. In this work, we observed that miR‐29b‐3p mimic could facilitate Bax expression, restrain Bcl2 expression and promote the activation of caspase‐3, which may contribute to the cell apoptosis. Moreover, the results from TUNEL staining showed that the pre‐administration of miR‐29b‐3p antagomir markedly decreased the chondrocyte apoptosis in cartilage tissue from OA rats.

The relationship between chondrocyte apoptosis and the occurrence and development of OA has not formed a conclusion. Zaitunnatakhin Zamli *et al*. demonstrated that chondrocyte apoptosis mainly occurred at the later stage of OA development when cells numbers in cartilage matrix markedly decreased, and the cartilage matrix was highly fibrillated, suggesting that chondrocyte apoptosis is a late event in OA 33. However, some other researchers believed that chondrocyte apoptosis was an early event in OA progress 14, 34. Despite this, accumulating studies have indicates that chondrocyte apoptosis plays an important role in the mechanisms of degeneration of articular cartilage tissues in OA 35. Thus, the mechanism of chondrocyte apoptosis may serve as targets for the treatment of OA. The risk factors that cause the apoptosis of chondrocyte in OA are varied, such as nitric oxide donors, Fas ligand, staurosporine, hypertrophy and lack of ECM or adhesion. A number of miRNAs were also implied to have functions in chondrocyte apoptosis in OA. It was identified that miR‐146a is involved in human chondrocyte apoptosis in response to mechanical injury and contribute to the pathogenesis of OA by modulating the VEGF and TGF‐β signalling pathway through the targeted inhibition of Smad4 in cartilage 36. In addition, miR‐34a was reported to influences chondrocyte apoptosis and proliferation through targeting the SIRT1/p53 during the pathogenesis of OA 37, and silencing of miR‐34a efficiently decreased rat chondrocyte apoptosis induced by IL‐1β 8. In this work, we demonstrated the loss of cartilage tissue caused by chondrocyte apoptosis was significantly inhibited by miR‐29b‐3p knock‐down using miR‐29b‐3p antagomir *in vivo*.

In addition to causing cartilage cell apoptosis, overexpression of miR‐29b‐3p was found to stimulate the expression of ECM degradation‐related enzymes, such as MMP‐1 and MMP‐13 in rat primary chondrocytes and SW‐1353 human chondrosarcoma cells, while down‐regulation of miR‐29b‐3p *in vivo* dramatically suppressed the production of MMP‐1, MMP‐13, ADAMTS‐5 and ADAMTS‐7, accompanied by the increase of the levels of ECM components, such as COMP, COL II, Aggrecan and COL X. Interestingly, the members of the miR‐29 family have attracted lots of attention in recent years due to its inhibition effect on ECM synthesis by targeting genes coding for many kinds of collagens, including and COL1A1, COL2A1, COL3A1, COL4A1, COL4A3, COL4A5, COL5A1, COL5A2, COL6A3 38. The inhibition effect of miR‐29b on collagen expression has been experimentally validated in many different experimental systems, including renal and cardiac fibrosis, systemic sclerosis and osteoclast differentiation, suggesting that miR‐29b may be a common mechanism in cells to regulate collagen production 39.

Progranulin is a growth factor, and involved in a variety of physiological processes, such as wound healing, inflammation, infection, tumourigenesis and neurodegeneration. PRGN is highly expressed in chondrocytes and play an important in cartilage formation and function. PGRN overexpression can promote chondrocyte proliferation, which is regulated by the (Erk) 1/2 pathway and Jun B transcription factor 40. PGRN can protect COMP from degradation by directly binding to ADAMTS‐7 and ADAMTS‐12 41. In addition, PGRN can suppress TNFα‐induced ADAMTS‐7 and ADAMTS‐12 production through binding to TNFR receptors. It was demonstrated that PGRN and its engineered derivative, Attstrin could restrain TNFα‐induced release of inflammatory and catabolic mediators, including MMP‐13, RUNX2, COX‐2 and iNOS in human chondrocytes *in vitro*, and promote the synthesis of ECM integrating components, such as Col 2 and Aggrecan 15, 42. Several studies have validated that PGRN knock‐out accelerated OA progress 16, 43, 44. Consistent with a previous study 41, we observed PGRN was overexpressed in OA in this research, which may be caused by the body's response to cartilage tissue injury.

Notably, it has been demonstrated that miR‐29b‐3p could directly regulate PGRN expression, which may be a possible pathogenesis of FTD. In this work, we identified a binding site for miRNA‐29b‐3p in the 3′ UTR of Rno PGRN mRNA. MiR‐29b‐3p mimic remarkably decreased luciferase expression through Rno PGRN, which was abolished by mutations in the 3′ UTR of Rno PGRN. In addition, miR‐29b‐3p mimic could significantly down‐regulate PGRN expression, while miR‐29b‐3p inhibitor promoted PGRN expression. Thus, we speculated that miR‐29b‐3p might induce chondrocyte apoptosis and cell cycle arrest by targeting PGRN, which was validated by the fact that recombinant PGRN or shPGRN‐mediated PGRN knock‐down reversed the effect of miR‐29b‐3p mimic on apoptosis, proliferation and wound closure of chondrosarcoma cells. As for why miR‐29b‐3p and GRN were both up‐regulated in OA sample, we speculated that GRN was not only modulated by miR‐29b‐3p, but also influenced by many other factors, such as TNF‐α and IL‐1β 45, which probably means there are some other mechanisms in the body to regulate GRN expression during OA. We believe that the increase of PGRN in OA samples is a comprehensive result of all various factors, and the final seemingly positive correlation may cover its complicated mechanism.

In conclusion, we demonstrated that miR‐29b‐3p‐induced chondrocyte apoptosis and cell cycle arrest by directly targeting PGRN in rat primary chondrocytes and SW‐1353 human chondrosarcoma cells. Knock‐down of miR‐29b‐3p using miR‐29b‐3p antagomir impeded the apoptosis of articular chondrocytes and the loss of cartilage in the knee joint of surgically induced rat OA model. MiR‐29b‐3p and PGRN may serve as potential targets for OA treatment.

## Conflict of interest

The authors confirm that there are no conflicts of interest.

## Author contribution

Jing Wang initiated and designed the study. Lingqiang Chen, Qin Li, Song Jin, Hongmei Zheng and Jun Lin participated in recruiting samples and/or performed laboratory studies. Fang He, Hong Zhang and Sha Ma periodically discussed the progression of project. Jian Mei and Juan Yu were responsible for data interpretation. Lingqiang Chen and Qin Li wrote the draft of manuscript. Jing Wang revised and formed the final manuscript. All authors have read and approved the final version of this submission.

## Supporting information


**Figure S1** Validation of the specificity of miR‐29b‐3p mimic and antagomir in viro and *in vivo*. (A‐B) The levels of other miR‐29 family members (miR‐29a‐3p and miR‐29c‐3p) were not influenced by miR‐29b‐3p mimic or miR‐29b‐3p inhibitor *in vitro*; (C‐D) The levels of miR‐29a‐3p and miR‐29c‐3p was not influenced by miR‐29b‐3p antagomir *in vivo*. *P < 0.05, **P < 0.01, compared with Sham group. NS: not significant.

**Figure S2** Knockdown of PGRN by the shRNA lentivirus that specifically targets GRN in wild type SW‐1353 cells, and the effect of miR‐29b‐3p mimic on PGRN in PGRN KD (knockdown) cells. (A‐C) The effect of lentiviral vector infection on mRNA (A) or protein (B, C) level of PGRN in SW‐1353 cells, *P < 0.05, **P < 0.01, compared with shNC group; (D‐F) The effect of miR‐29b‐3p mimic on PGRN expression in PGRN KD cells. *P < 0.05, **P < 0.01, compared with NC group in wild type or PGRN KD cells; NS: not significant.

**Table S1** Primers used in qRT‐PCR
