# Supplementary material for: MiR‐29b‐3p promotes chondrocyte apoptosis and facilitates the occurrence and development of osteoarthritis by targeting PGRN
Source: J Cell Mol Med. 2017 Jun 13;21(12):3347–59. doi: 10.1111/jcmm.13237 (PMC5706578; doi:10.1111/jcmm.13237)
Supplement: Supplementary file 3 — Table S1 Primers used in qRT‐PCR [file JCMM-21-3347-s003.doc]

**Table S1** Primers used in qRT-PCR

| Gene | RT (5’→3’) | Forward (5’→3’) | Reverse (5’→3’) |
| --- | --- | --- | --- |
| miR-29a-3p | GTCGTATCCAGTGCAGGGTCCGAGGTATTCGCACTGGATACGACaatccg | UAGCACCUACUGAAAU | GTGCAGGGTCCGAGGT |
| miR-29b-3p | GTCGTATCCAGTGCAGGGTCCGAGGTATTCGCACTGGATACGACaacact | UAGCACCAUUUGAAAUC | GTGCAGGGTCCGAGGT |
| miR-29c-3p | GTCGTATCCAGTGCAGGGTCCGAGGTATTCGCACTGGATACGACtaaccg | UAGCACCAUUUGAAAU | GTGCAGGGTCCGAGGT |
| U6 | AACGCTTCACGAATTTGCGT | GCTTCGGCAGCACATATAC | AACGCTTCACGAATTTGCGT |
| hsa-GRN | random hexamer | CCTGGACCCCGGAGGAGC | ACGGTAAAGATGCAGGAGTGG |
| hsa-COL2A1 | random hexamer | GCACCTGCAGAGACCTGAAAC | GCAAGTCTCGCCA GTCTCCA |
| hsa-COL10A1 | random hexamer | CATAAAAGGCCCACTACCCAAC | ACCTTGCTCTCC TCTTACTGC |
| hsa-β-actin | random hexamer | CATGTACGTTGCTATCCAGGC | CTCCTTAATGTCACGC ACGAT |
| rno-GRN | random hexamer | CCAGGTGCTCTTCTCGATCC | AAGTGTACAAACTTTATTGGAGCA |
| rno-COL2A1 | random hexamer | GCCAGGATGCCCGAAAATTAG | GGCTGCAAAGTTTCCTCCAC |
| rno-COL10A1 | random hexamer | GACCCAGGAAAGCCAGGTG | AGCACTGACAAGAGGCATCC |
| rno-β-actin | random hexamer | TGTCACCAACTGGGACGATA | AACACAGCCTGGATGG CTAC |
